# Associations between benign paroxysmal positional vertigo and seven mental disorders: a two-sample Mendelian randomization study

**DOI:** 10.3389/fneur.2024.1310026

**Published:** 2024-04-09

**Authors:** Shihan Liu, Lingli Zhang, Dan Deng, Wenlong Luo

**Affiliations:** ^1^Department of Otorhinolaryngology, The Second Affiliated Hospital of Chongqing Medical University, Chongqing, China; ^2^Department of Otorhinolaryngology, Central Hospital Affiliated to Chongqing University of Technology, Chongqing, China; ^3^Department of Eye and ENT, Chongqing Maternal and Child Health Care Hospital, Chongqing, China

**Keywords:** Mendelian randomization, benign paroxysmal positional vertigo, mental disorders, neuroticism, mood swings

## Abstract

**Background:**

The association between benign paroxysmal positional vertigo (BPPV) and various mental disorders is still controversial. This study used the Mendelian randomization (MR) method to clarify the correlation between BPPV and seven mental disorders (bipolar disorder, depression, anxiety disorder, schizophrenia, suicidality, neuroticism, and mood swings) to aid in the exploration of BPPV complications and prevention and early treatment of mental disorders.

**Methods:**

The datasets for BPPV and seven mental disorders were obtained from genome-wide association studies (GWASs). Two-sample MR was used to analyze the correlation between exposure (BPPV) and various outcomes (bipolar disorder, depression, anxiety disorder, schizophrenia, suicidality, neuroticism, and mood swings). A reverse MR study was also performed. The inverse variance weighting (IVW) method, the MR–Egger method, the simple mode method, the weighted mode method, and the weighted median method were selected.

**Results:**

The MR analysis and the reverse MR analysis results did not reveal significant associations between BPPV and bipolar disorder, depression, anxiety disorder, schizophrenia, suicidal tendencies, neuroticism, and mood swings. Interestingly, neuroticism (IVW: OR = 1.142, 95% CI: 1.059–1.231, *P* = 0.001; P-MR-PRESSO adjustment = 0.0002) and mood swings (IVW: OR = 3.119, 95% CI: 1.652–5.884, *P* = 0.0004) may have a significant association with BPPV. After MR-PRESSO adjustment, there was no horizontal pleiotropy or heterogeneity, and a significant association between neuroticism, mood swings, and BPPV has still been suggested.

**Conclusion:**

We conducted MR analysis on genetic data from European populations and discovered a causal relationship between BPPV and the seven mental disorders. Our research findings suggest that BPPV may not have a significant causal relationship with bipolar disorder, depression, anxiety disorder, schizophrenia, or suicidal tendencies. However, neuroticism and mood swings may be risk factors for BPPV.

## 1 Introduction

Benign paroxysmal positional vertigo (BPPV) is the most common cause of vertigo, and 24.1% of patients with dizziness/vertigo have BPPV (1). The underlying mechanism of BPPV may be the displacement of degenerate otoliths into the semicircular canal, resulting in increased sensitivity to head movement, which induces paroxysmal positional vertigo (2). The lifetime incidence of BPPV is as high as 2.4% (3), and BPPV seriously affects the quality of life in affected individuals (4), increases their risk of falls, and reduces their walking speed (5). BPPV has caused a significant medical burden worldwide (6). Therefore, exploring the impact of BPPV on the incidence of other diseases would be highly helpful for informing personalized treatment and improving patient prognosis.

At present, increased attention has been given to mental disorders worldwide. Approximately one in five people experience a common mental disorder in a year (7). Mental disorders lead to a serious decline in the participation rate of affected individuals in the social labor force, and the high cost of treatment seriously affects their quality of life in the later stages of the illness (8, 9). Multiple diseases or behaviors are thought to contribute to an increased prevalence of mental disorders (10–12). Therefore, identifying the risk factors for mental disorders could facilitate early intervention for individuals affected, thereby reducing the impact of mental disorders on both patients and society.

Although vertigo caused by BPPV can be resolved by the implementation of repeated canalith repositioning procedure (CRP), symptoms of vertigo and positional nystagmus in the patient often return (13). However, some studies have suggested that the clinical features of paroxysmal vertigo may induce various mental disorders in patients with BPPV. At present, whether BPPV can increase the risk of various mental disorders in patients is still controversial, and related studies are rare. A cohort study from Taiwan, China, suggested that chronic stress due to paroxysmal vertigo may increase the risk of BPPV-related suicide (14). A survey of the incidence of BPPV in all patients with mood disorders in Korea revealed that mood disorders may be significantly associated with BPPV (15). A recent meta-analysis suggested that BPPV may increase the risk of anxiety, but no significant association between BPPV and depression was found. There were few relevant studies included in this meta-analysis, and the sample size was small; therefore, further research is needed to determine the associations between BPPV and anxiety and depression (16). Similar to anxiety and depression, bipolar disorder and schizophrenia are also common mental disorders (17), and no relevant studies have explored the associations between bipolar disorder and schizophrenia and BPPV. A lower neuroticism score and stable emotions play a certain role in mental health (18, 19). However, recurrent progression of vertigo may lead to greater neuroticism and mood swings in patients (20).

Many studies have explored the association between mental disorders and diseases through Mendelian randomization (MR) (11, 21). MR is used to clarify the association between two traits. Genetic variants are included as instrumental variables. Single-nucleotide polymorphisms (SNPs) are identified from independent genome-wide association study (GWAS) datasets and are subjected to association analysis as instrumental variables (22). The advantages of MR include avoiding the limitations of traditional observational research and eliminating the interference of various confounding factors in the study as much as possible so that the research results have greater credibility. MR studies have improved the statistical power to infer causal relationships between diseases (23). This study aimed to analyze the relationship between BPPV and seven mental disorders (bipolar disorder, depression, anxiety disorder, schizophrenia, suicidality, neuroticism, and mood swings) by using the MR method to clarify whether there is a correlation between BPPV and seven mental disorders. Neuroticism and the presence of mood swings are considered risk factors for mental disorders; therefore, these factors were included in this study to explore the correlation between BPPV and neuroticism and mood swings. The association between BPPV and mental disorders is clarified to improve the timeliness and targeting of the prevention and treatment of both conditions.

## 2 Methods

### 2.1 Data sources

In this study, a two-sample MR analysis was used to analyze the relationship between exposure (BPPV) and various outcomes (bipolar disorder, depression, anxiety disorder, schizophrenia, suicidality, neuroticism, and mood swings). Reverse MR was applied to analyze the correlation between exposure (bipolar disorder, depression, anxiety disorder, schizophrenia, suicidality, neuroticism, and mood swings) and an outcome (BPPV). The GWAS datasets used in this study were all obtained from the IEU GWAS database (https://gwas.mrcieu.ac.uk/), from which the datasets for BPPV and bipolar disorder, depression, anxiety, schizophrenia, suicidality, neuroticism, and mood swings were selected. The BPPV dataset was collected from the FinnGen database, which includes genomic and health data collected from 500,000 Finnish biobanks to determine the genetic basis of the disease. The IEU database has obtained the BPPV dataset from the FinnGen database R5 version. The diagnosis criteria in the FinnGen database are based on the Tenth Revision of the International Statistical Classification of Diseases and Related Health Problems (ICD-10). The diagnosis of BPPV requires meeting the diagnostic criteria with the code H81.1 according to the ICD-10. Depression, anxiety disorders, suicidality, neuroticism, and mood swing-related datasets were collected from the UK Biobank, which includes genetic information obtained from more than 500,000 participants from all over the UK. The bipolar disorder and schizophrenia dataset was derived from a GWAS database of patients with bipolar disorder and schizophrenia (24, 25). Detailed information on the GWAS data sources used in our study is provided in Table 1.

**Table 1:** The GWAS data sources.

**Phenotype**	**Data source**	**PMID**	**Cases**	**Controls**	**Sample size**	**Ancestry**
BPPV	FinnGen		3,834	209,582	213,416	European
Bipolar disorder	Stahl, E et al.	31043756	20,352	31,358	51,710	European
Depression	Ben Elsworth et al.		26,595	436,338	462,933	European
Anxiety disorders	Ben Elsworth et al.		6,410	456,523	462,933	European
Schizophrenia	Trubetskoy V et al.	35396580	52,017	75,889	127,906	European
Suicidality	Neale laboratory		2,658	2,275	4,933	European
Neuroticism	Ben Elsworth et al.				374,323	European
Mood swings	Ben Elsworth et al.		204,412	247,207	451,619	European

BPPV, benign paroxysmal positional vertigo.

### 2.2 Selection of instrumental variables

The SNPs were selected from the GWAS dataset based on the following conditions: 1. The significance in genome-wide studies to prevent the inclusion of fewer SNPs (*P* < 5^*^10^−6^ was selected as the screening criterion). 2. No linkage disequilibrium was detected between any of the SNPs to preserve SNP independence (r2 <0.001 and 10,000 kb). 3. SNPs with an F-statistic <10 were excluded as they were considered weak instrumental variables. Plus-strand allele inference was then attempted using palindromic allele frequencies.

### 2.3 Mendelian randomization analysis

The inverse variance weighting (IVW), MR–Egger, simple mode, weighted mode, and weighted median methods were used for data evaluation. IVW obtained a total estimate of the effect of exposure on the outcome by combining the causal estimate of the Wald ratio for each IV, and IVW was used as the primary analysis method (26). The non-zero intercept values shown by the MR–Egger method were mainly used to examine horizontal pleiotropy (27). The weighted median gives an accurate estimate based on the assumption that at least 50% of IVs are effective (28). The simple mode, weighted mode, and weighted median methods were mainly used to verify the reliability and stability of the results. Causality was assessed using the odds ratio (OR) and 95% confidence interval (95% CI) to determine the significance. To strengthen the reliability of this study, the significance was set at 0.05/7 (0.007) according to the Bonferroni correction method.

The MR–Egger method was used to obtain intercept values to evaluate horizontal pleiotropy. The *Q*-statistic from Cochran's IVW was then used to investigate the impact of heterogeneity. The results of pleiotropic and heterogeneous MR-PRESSO analysis were obtained to remove outlier SNPs from the group and recalculate the MR results.

MR analysis was performed using the TwoSampleMR package in R version 4.2.3 (http://www.r-project.org) (29). The TwoSampleMR package enables online analysis of the association between exposure and outcome datasets through the IEU database.

## 3 Results

### 3.1 The results of MR analysis between BPPV and seven mental disorders

**Figure 1:**
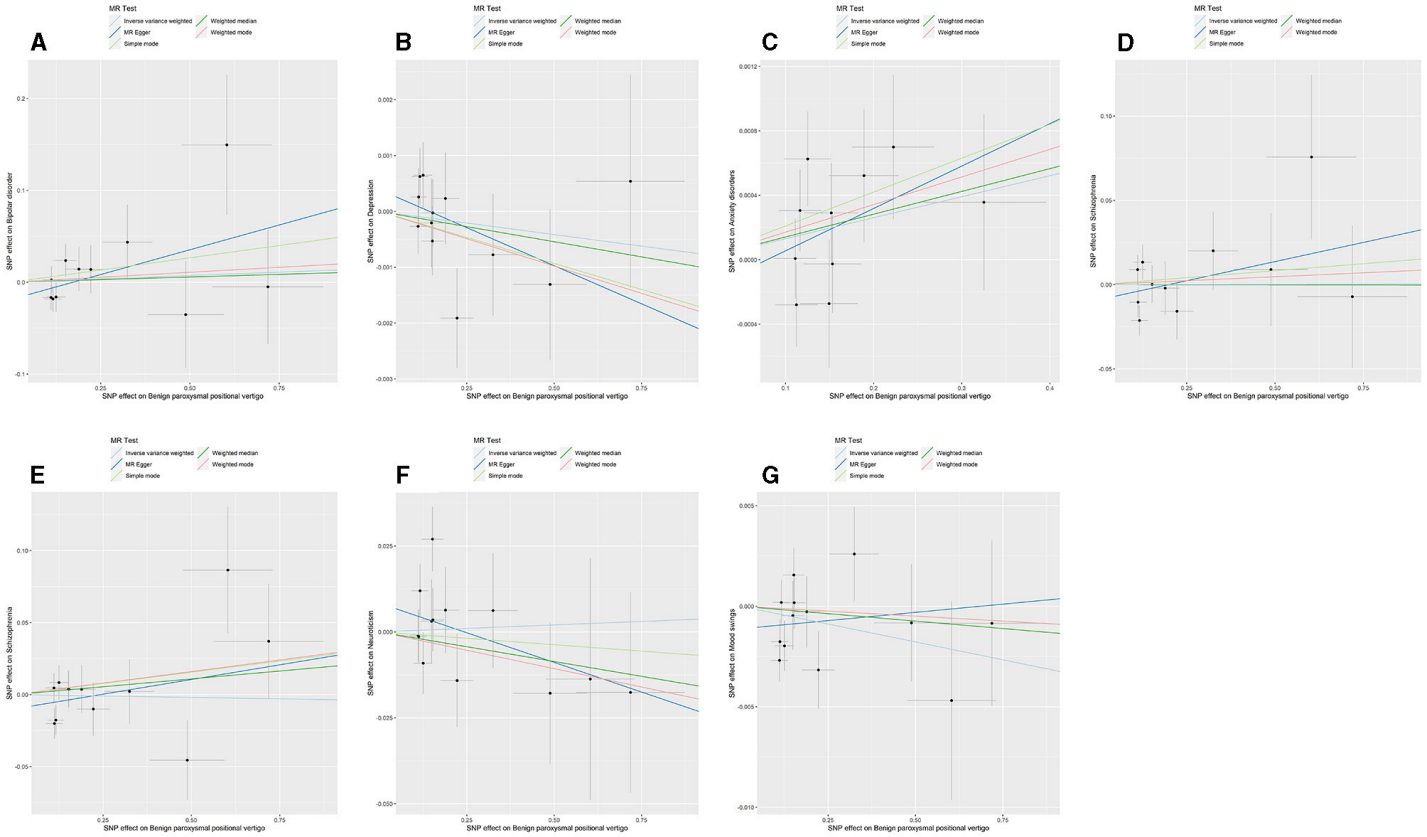
Summary view of the MR images derived from the IVW, MR–Egger, simple mode, weighted median, and weighted mode methods. **(A)** The MR analysis results for BPPV and bipolar disorder. **(B)** The MR analysis results for BPPV and depression. **(C)** The MR analysis results for BPPV and anxiety disorders. **(D)** The MR analysis results for BPPV and schizophrenia. **(E)** The MR analysis results for BPPV and suicidality. **(F)** The MR analysis results for BPPV and neuroticism. **(G)** The MR analysis results for BPPV and mood swings.

**Figure 2:**
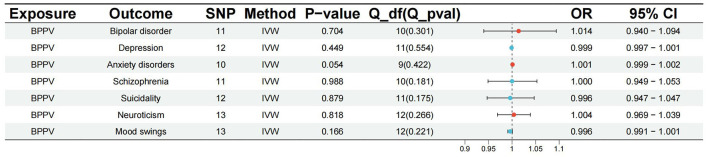
The results of the MR analysis between BPPV and mental disorders.

The *p*-value of <5^*^10^−6^ was selected as the screening criterion for BPPV-related SNPs. After screening based on the screening criteria, MR analysis was performed, and the F-statistics of the SNPs included in the analysis were all found to be >10, indicating that they were all strong instrumental variables (Supplementary material). All heterogeneity analyses showed results that *p* > 0.05, which suggested that there was no heterogeneity in the results. No horizontal pleiotropy was found in any of the MR–Egger analyses (*P* > 0.05). The results suggested that there was no significant association between BPPV and bipolar disorder (IVW: OR = 1.014, 95% CI: 0.940–1.094, *P* = 0.704), depression (IVW: OR = 0.999, 95% CI: 0.997–1.001, *P* = 0.449), anxiety disorders (IVW: OR = 1.001, 95% CI: 0.999–1.002, *P* = 0.054), schizophrenia (IVW: OR = 1.000, 95% CI: 0.949–1.053, *P* = 0.988), suicidality (IVW: OR = 0.996, 95% CI: 0.947–1.047, *P* = 0.879), neuroticism (IVW: OR = 1.004, 95% CI: 0.969–1.039, *P* = 0.818), and mood swings (IVW: OR = 0.996, 95% CI: 0.991–1.001, *P* = 0.166) (Figures 1, 2). The detailed analysis results are shown in Table 2.

**Table 2:** Results of the MR analysis of BPPV and mental disorders.

**Exposure**	**Outcome**	**SNP**	**Method**	**OR**	**95% CI**	***P*-value**	**Q_df (Q_pval)**
BPPV	Bipolar disorder	11	MR Egger	1.113	0.968–1.281	0.165	9 (0.404)
			Weighted median	1.011	0.916–1.116	0.822	
			IVW	1.014	0.940–1.094	0.704	10 (0.301)
			Simple mode	1.054	0.893–1.245	0.543	
			Weighted mode	1.021	0.866–1.205	0.802	
BPPV	Depression	12	MR Egger	0.997	0.992–1.001	0.246	10 (0.554)
			Weighted median	0.998	0.995–1.001	0.478	
			IVW	0.999	0.997–1.001	0.449	11 (0.554)
			Simple mode	0.998	0.993–1.002	0.465	
			Weighted mode	0.998	0.994–1.001	0.345	
BPPV	Anxiety disorders	10	MR Egger	1.002	0.998–1.007	0.291	8 (0.361)
			Weighted median	1.001	0.999–1.003	0.119	
			IVW	1.001	0.999–1.002	0.054	9 (0.422)
			Simple mode	1.002	0.999–1.004	0.186	
			Weighted mode	1.001	0.999–1.004	0.212	
BPPV	Schizophrenia	11	MR Egger	1.046	0.944–1.159	0.406	9 (0.190)
			Weighted median	0.999	0.937–1.066	0.994	
			IVW	1.000	0.949–1.053	0.988	10 (0.181)
			Simple mode	1.016	0.915–1.129	0.763	
			Weighted mode	1.009	0.926–1.099	0.835	
BPPV	Suicidality	12	MR Egger	1.041	0.947–1.145	0.418	10 (0.194)
			Weighted median	1.022	0.962–1.085	0.476	
			IVW	0.996	0.947–1.047	0.879	11 (0.175)
			Simple mode	1.031	0.933–1.139	0.552	
			Weighted mode	1.032	0.934–1.140	0.540	
BPPV	Neuroticism	13	MR Egger	0.966	0.905–1.030	0.321	11 (0.329)
			Weighted median	0.983	0.940–1.027	0.443	
			IVW	1.004	0.969–1.039	0.818	12 (0.266)
			Simple mode	0.993	0.927–1.062	0.832	
			Weighted mode	0.979	0.924–1.036	0.475	
BPPV	Mood swings	13	MR Egger	1.002	0.992–1.011	0.742	11 (0.263)
			Weighted median	0.999	0.992–1.004	0.647	
			IVW	0.996	0.991–1.001	0.166	12 (0.221)
			Simple mode	0.999	0.988–1.009	0.857	
			Weighted mode	0.999	0.989–1.008	0.838	

BPPV, Benign paroxysmal positional vertigo.

### 3.2 The results of MR analysis between seven mental disorders and BPPV

**Figure 3:**
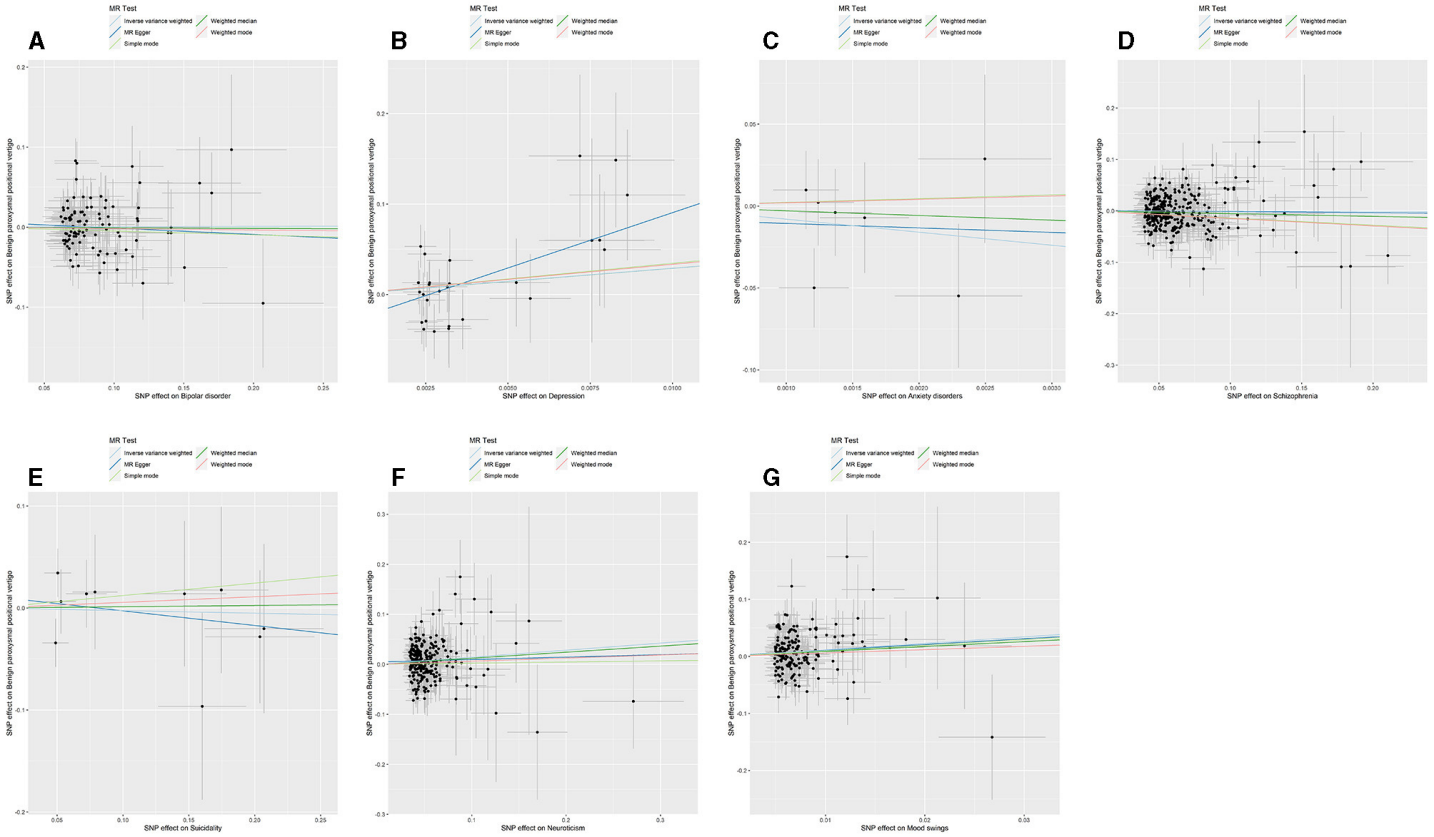
Summary view of the MR images derived from the IVW, MR–Egger, simple mode, weighted median, and weighted mode methods. (MR-PRESSO adjustment) **(A)** The MR analysis results for bipolar disorder and BPPV. **(B)** The MR analysis results for depression and BPPV. **(C)** The MR analysis results for anxiety disorders and BPPV. **(D)** The MR analysis results for schizophrenia and BPPV. **(E)** The MR analysis results for suicidality and BPPV. **(F)** The MR analysis results for neuroticism and BPPV. **(G)** The MR analysis results for mood swings and BPPV.

**Figure 4:**
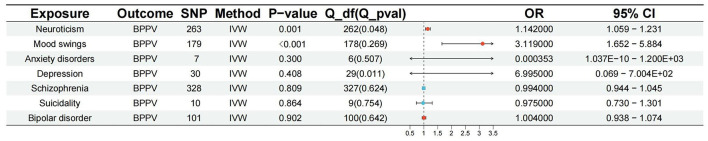
The results of the MR analysis between mental disorders and BPPV.

The *p*-value of <5^*^10^−6^ was selected as the screening criterion for seven mental disorder-related SNPs. After screening based on the criteria, MR analysis was performed, and the *F*-statistics of the SNPs included in the analysis were all found to be >10, indicating strong instrumental variables (Supplementary material). No significant association was found in the reverse MR of bipolar disorder (IVW: OR = 1.004, 95% CI: 0.938–1.074, *P* = 0.902), depression (IVW: OR = 6.995, 95% CI: 0.069–7.004E+02, *P* = 0.408, P-MR-PRESSO adjustment = 0.147), anxiety (IVW: OR = 3.529E-04, 95% CI: 1.037E-10-1.200E+03, *P* = 0.300), schizophrenia (IVW: OR = 0.994, 95% CI: 0.944–1.045, *P* = 0.809), suicidality (IVW: OR = 0.975, 95% CI: 0.730–1.301, *P* = 0.864), and BPPV. Neuroticism (IVW: OR = 1.142, 95% CI: 1.059–1.231, *P* = **0.001**; *P-MR-PRESSO adjustment* = **0.0002**) and mood swings (IVW: OR = 3.119, 95% CI: 1.652–5.884, *P* = **0.0004**) were significantly associated with BPPV. Horizontal pleiotropy and heterogeneity were detected in the reverse MR analysis of patients with depression and BPPV, and heterogeneity was detected in the inverse variance MR analysis of patients with neuroticism and BPPV. MR analysis was performed again after MR-PRESSO adjustment, and the results showed a lack of horizontal pleiotropy and heterogeneity (Figures 3, 4). The detailed analysis results are shown in Table 3.

**Table 3:** Results of the MR analysis of mental disorders and BPPV.

**Exposure**	**Outcome**	**SNP**	**Method**	**OR**	**95% CI**	***P*-value**	**Q_df (Q_pval)**	***P*-value (MR-PRESSON after adjustment)**
Bipolar disorder	BPPV	101	MR Egger	0.924	0.675–1.264	0.622	99 (0.623)	
			Weighted median	0.992	0.898–1.095	0.882		
			IVW	1.004	0.938–1.074	0.902	100 (0.642)	
			Simple mode	0.953	0.732–1.240	0.723		
			Weighted mode	0.983	0.764–1.263	0.892		
Depression	BPPV	30	MR Egger	3.419E+07	3.733E+3–3.131E+12	**0.006**	28 (0.097)	0.028
			Weighted median	17.253	0.089–3.318E+03	0.288		0.293
			IVW	6.995	0.069–7.004E+02	0.408	29 (0.011)	0.147
			Simple mode	39.385	4.942E-04–3.139E+06	0.529		0.543
			Weighted mode	39.385	2.188E-03–7.090E+05	0.468		0.502
Anxiety disorders	BPPV	7	MR Egger	0.065	4.480E-28–9.384E+24	0.933	5 (0.385)	
			Weighted median	0.058	2.737E-11–1.214E+08	0.794		
			IVW	3.529E-04	1.037E-10–1.200E+03	0.300	6 (0.507)	
			Simple mode	9.836	3.593E-12–2.691E+13	0.881		
			Weighted mode	7.861	2.101E-12–2.941E+13	0.894		
Schizophrenia	BPPV	328	MR Egger	0.978	0.809–1.181	0.819	326 (0.609)	
			Weighted median	0.948	0.878–1.023	0.171		
			IVW	0.994	0.944–1.045	0.809	327 (0.624)	
			Simple mode	0.870	0.667–1.134	0.306		
			Weighted mode	0.864	0.672–1.109	0.253		
Suicidality	BPPV	10	MR Egger	0.866	0.497–1.509	0.626	8 (0.690)	
			Weighted median	1.012	0.684–1.496	0.951		
			IVW	0.975	0.730–1.301	0.864	9 (0.754)	
			Simple mode	1.130	0.648–1.968	0.677		
			Weighted mode	1.057	0.630–1.770	0.839		
Neuroticism	BPPV	263	MR Egger	1.042	0.760–1.427	0.798	261 (0.046)	0.757
			Weighted median	1.126	1.012–1.251	0.028		0.026
			IVW	1.142	1.059–1.231	**0.001**	262 (0.048)	**0.0002**
			Simple mode	1.034	0.730–1.463	0.851		0.902
			Weighted mode	1.066	0.768–1.480	0.701		0.716
Mood swings	BPPV	179	MR Egger	2.713	0.195–37.608	0.458	177 (0.252)	
			Weighted median	2.357	0.928–5.979	0.071		
			IVW	3.119	1.652–5.884	**0.0004**	178 (0.269)	
			Simple mode	2.648	0.145–48.330	0.512		
			Weighted mode	1.795	0.132–24.352	0.661		

BPPV, Benign paroxysmal positional vertigo. Bold values indicates that *P*-values are significant.

## 4 Discussion

In this study, the MR method was used to assess the association between BPPV and seven mental disorders. The results showed that BPPV was not significantly associated with bipolar disorder, depression, anxiety disorders, schizophrenia, or suicidality. Reverse MR analysis indicated that bipolar disorder, depression, anxiety, schizophrenia, and suicidality were not significantly associated with BPPV, while higher neuroticism scores and mood swings may promote the occurrence and development of BPPV. Analyses of horizontal pleiotropy and heterogeneity after MR-PRESSO adjustment did not reveal significant differences, which suggests the reliability of the results.

In related studies analyzing patients with BPPV in Korea, it was found that the risk of developing mood disorders in BPPV patients was significantly greater than that in healthy people (15). The degree of anxiety and depression may reflect the probability of residual dizziness after canalith repositioning (30). At present, the associations between BPPV and anxiety and depression have been studied the most. A recent meta-analysis of 23 studies and 2,902 patients showed that there was a significant association between BPPV and anxiety, but the association between BPPV and depression still needs to be further studied (16). Yang et al. conducted an analysis of 72,569 patients with peripheral vestibular disorders and 217,707 healthy controls in Taiwan and reported that suicidal attempts were strongly associated with BPPV, Meniere's disease, and vestibular neuritis; however, due to the uncertainty of other suicide risk factors, the association between these conditions needs to be further studied (14), and other studies have shown results similar to those in our analyses. Kalderon et al. analyzed the clinical data of 18 patients with BPPV and 18 healthy controls and reported that there may be no difference in anxiety between patients with BPPV and healthy controls (31). In our research, no relevant clinical studies on BPPV or bipolar disorder or schizophrenia were found, and the association between BPPV and the relevance of bipolar disorder and schizophrenia may require further exploration. Psychological distress has been shown to predict the severity of vestibular dysfunction to a certain extent (32). Neuroticism and mood swings, which are common psychological factors (33), may also have a certain effect on BPPV. Several clinical studies have confirmed our results from other perspectives (20, 34, 35). Our results are inconsistent with the results of several clinical analyses, possibly due to the lack of reliability of the results due to the unmeasured confounding factors that often appear in clinical studies of mental disorders or BPPV. Therefore, the results of clinical studies cannot fully reflect the association between these diseases. We used the MR method at the level of genetic analysis to determine the relationship between the two parameters (mental disorders and BPPV), ruling out various confounding factors, and thus improved the reliability of the results (36).

Due to the influence of various factors on the mechanism of BPPV, there may be no significant association between several mental disorders and this disease. Neuroticism and mood swings are more likely to be the risk factors for BPPV compared to other mental disorders. However, the mechanism by which neuroticism and mood swings, as common psychological distress factors, affect the occurrence and development of BPPV is still unclear, and local inflammation due to abnormal psychology could promote the development of BPPV (37, 38). Psychological stress can trigger a systemic stress response, leading to an inflammatory reaction. This regulation of an inflammatory reaction may serve a protective function in the short term, but sustained chronic inflammation stimulation may affect the functioning of the balance receptors in the inner ear, ultimately promoting the development of BPPV (39). Additionally, neuroticism and mood swings may enhance neural network activity, thereby affecting patients' visual balance control (40). Stable visual perception is crucial for individuals with BPPV (41). Further exploration of the relevant mechanisms is needed in the future. A deeper understanding of these mechanisms will aid in the development of more effective treatment strategies and preventive measures for BPPV.

Although our results suggest that there is no significant association between BPPV and five mental disorders (bipolar disorder, depression, anxiety disorder, schizophrenia, and suicidality), BPPV may have some influence on the occurrence and development of the five mental disorders. The underlying mechanisms of BPPV and mental disorders are complex. It is possible that long-term repeated harmful physical stimuli, such as chronic pain, may lead to emotional changes in patients, which may induce mental disorders (42). It has been suggested that somatic imbalance, spatial orientation disorder, nausea, and vomiting caused by recurrent vertigo attacks lead to secondary psychological distress (43). It has also been hypothesized that the cerebellar and vestibular systems play complementary roles in emotion regulation and that long-term maladaptation to the environment may lead to anxiety and depression (44, 45). Hemispheric lateralization may link vestibular systems to systems that process emotions (46). The chronic physical stress caused by BPPV will also continue to affect the hypothalamic–pituitary–adrenal (HPA) axis (47), and disorders of the HPA axis may affect mood in individuals (48). The exploration of the mechanisms underlying the correlation between neuroticism and mood swings and BPPV merits further study because of the association between BPPV and mental disorders, which may be significant for guiding future research on the underlying mechanisms of the associations between psychological states and BPPV.

To date, no MR study has examined the association between BPPV and mental disorders. We used MR analysis in this study to avoid the bias caused by confounding factors and sample size difficulties that occur in traditional clinical research. The reliability and accuracy of the study were improved. MR analysis strengthened the causal relationship and reduced the probability of confounding and reverse causality. This study has some limitations. Because the datasets were obtained from a public database and the patients were of European ancestry, the results of this study were not necessarily generalizable to other regions or ethnic groups. Although we did not find horizontal pleiotropy after adjustment for MR-PRESSO, we cannot completely rule out that horizontal pleiotropy affected the generalizability of our results. Since the datasets used in this study were obtained from a public database, we cannot classify the sample population by age and sex or analyze their correlation more precisely. Although the findings of the study established a causal relationship between BPPV and neuroticism and mood swings, future research should involve additional design interventions targeting the risk factors for BPPV to aid in the development of better prevention for the recurrence of BPPV.

## 5 Conclusion

In summary, the results of the two-sample MR analysis revealed that BPPV was not significantly associated with five mental disorders (bipolar disorder, depression, anxiety disorders, schizophrenia, and suicidality). Neuroticism and mood swings are more likely to be the risk factors for BPPV. Therefore, we need to pay more attention to the psychological distress in BPPV patients, and we need to treat BPPV and prevent its recurrence. The association between BPPV and mental disorders is clarified to improve the early prevention and treatment of mental disorders and BPPV in clinical research. The findings of this study will help to improve the comprehensive medical management of patients with mental disorders and BPPV in clinical practice and contribute to further revealing the underlying mechanisms of mental disorders and BPPV.

## Data availability statement

The original contributions presented in the study are included in the article/Supplementary material, further inquiries can be directed to the corresponding author.

## Ethics statement

Ethical review and approval was not required for the study on human participants in accordance with the local legislation and institutional requirements. Written informed consent from the patients/participants or patients/participants' legal guardian/next of kin was not required to participate in this study in accordance with the national legislation and the institutional requirements.

## Author contributions

SL: Conceptualization, Data curation, Software, Visualization, Writing – original draft. LZ: Investigation, Methodology, Project administration, Supervision, Writing – review & editing. DD: Project administration, Supervision, Visualization, Writing – review & editing, Conceptualization, Funding acquisition. WL: Project administration, Supervision, Visualization, Writing – review & editing.
