# Supplementary material for: Associations between benign paroxysmal positional vertigo and seven mental disorders: a two-sample Mendelian randomization study
Source: Front Neurol. 2024 Apr 9;15:1310026. doi: 10.3389/fneur.2024.1310026 (PMC11035833; doi:10.3389/fneur.2024.1310026)
Supplement: Supplementary file 1 [file Data_Sheet_1.docx]

**MR analysis code**

##GWAS ID: BPPV(finn-b-H8_BPV)；

##GWAS ID:Bipolar disorder(ieu-b-41);

##GWAS ID:Depression(ukb-b-12064);

##GWAS ID:Anxiety disorders(ukb-b-17243);

##GWAS ID:Schizophrenia(ieu-b-5102);

##GWAS ID:Suicidality(ukb-d-20483);

##GWAS ID:Neuroticism(ukb-b-4630);

##GWAS ID:Mood swings(ukb-b-14180);

library(TwoSampleMR)

TL<-extract_instruments(outcomes = 'finn-b-H8_BPV', p1 =5e-06)#Extracting Exposure data

write.csv(TL,'telomere length.csv')

outcome_dat<-extract_outcome_data(snps = TL$SNP,outcomes = 'ieu-b-41' ,proxies = F)#Extraction OF Outcome data

write.csv(outcome_dat,'outcome.csv')

outcome_dat<-read.csv('outcome.csv',header = T)

dat<-harmonise_data(TL,outcome_dat,action = 2) #Allele alignment, palindromic sequence

write.csv(dat,'dat.csv')

dat<-read.csv('dat.csv',header = T)

dat$EAF2 <- (1 - dat$eaf.exposure)

dat$MAF <- pmin(dat$eaf.exposure, dat$EAF2)

PVEfx <- function(BETA, MAF, SE, N){

pve <- (2*(BETA^2)*MAF*(1 - MAF))/((2*(BETA^2)*MAF*(1 - MAF)) + ((SE^2)*2*N*MAF*(1 - MAF)))

return(pve)

}

dat$PVE <- mapply(PVEfx, dat$beta.exposure, dat$MAF, dat$se.exposure, N = dat$samplesize.exposure)

dat$FSTAT <- ((dat$samplesize.exposure - 1 - 1)/1)*(dat$PVE/(1 - dat$PVE)) #F-statistic

write.csv(dat,'datF.csv')

dat<-read.csv('datF.csv',header = T)

results<-mr(dat) #MR Computing

write.csv(results,'results.csv')

OR <-generate_odds_ratios(results) #OR

write.csv(OR,'OR.csv')

#Import your filtered file

DAT<-read.csv('datF.csv',header = T)

heterogeneity <- mr_heterogeneity(DAT) #heterogeneity

heterogeneity

write.csv(heterogeneity,'heterogeneity.csv')

pleio <- mr_pleiotropy_test(DAT)#pleiotropy

pleio

write.csv(pleio,'pleio.csv')

#MRPRESSO

library(MRPRESSO)

mr_presso(BetaOutcome = 'beta.outcome',

BetaExposure = 'beta.exposure',

SdOutcome = 'se.outcome',

SdExposure = 'se.exposure',

data = DAT, OUTLIERtest = TRUE,

DISTORTIONtest = TRUE, SignifThreshold = 0.05, NbDistribution = 5000, seed = NULL)

#visualization

library(TwoSampleMR)

results<-read.csv('results.csv',header = T)

dat <- read.table("datF.csv", sep=",", header=T)

DAT<-read.csv('datF.csv',header = T)

single <- mr_leaveoneout(DAT)

mr_leaveoneout_plot(single)

p<-mr_scatter_plot(results,DAT)

png(filename = 'scatter2.tif',width = 2500,height = 3000,res = 350)

print(p)

dev.off()
